# Perceived risks and amelioration of harm in research using mobile technology to support antiretroviral therapy adherence in the context of methamphetamine use: a focus group study among minorities living with HIV

**DOI:** 10.1186/s12954-020-00384-1

**Published:** 2020-06-11

**Authors:** Elizabeth C. Pasipanodya, Maulika Kohli, Celia B. Fisher, David J. Moore, Brenda Curtis

**Affiliations:** 1 Rehabilitation Research Center, Santa Clara Valley Medical Center, San Jose, CA 95128 USA; 2 San Diego State University/University of California, San Diego Joint Doctoral Program in Clinical Psychology, San Diego, CA 92093 USA; 3 HIV Neurobehavioral Research Program, University of California, San Diego, CA 92103 USA; 4 Fordham University Center for Ethics Education, Fordham University, New York, NY 10023 USA; 5 Technology and Translational Research Unit, National Institute of Drug and Alcohol Abuse Intramural, Baltimore, MD 21224 USA

**Keywords:** HIV, ART adherence, Methamphetamine, mHealth, Participatory design, Bioethics

## Abstract

**Background:**

Methamphetamine use poses a barrier to antiretroviral therapy (ART) adherence. Black and Hispanic men who have sex with men living with HIV (PLWH) shoulder much of the health burden resulting from the methamphetamine and HIV syndemic. Smartphones are nearly ubiquitous in the USA and may be promising vehicles for delivering interventions for ART adherence and drug use cessation. However, the acceptability of using applications to collect sensitive information and deliver feedback in this population has not been adequately explored.

**Objective:**

This study examined minority PLWH’s appraisals of the risks of participating in smartphone-based research to promote ART adherence in the context of methamphetamine use and explored their views on appropriate steps to mitigate perceived risks of participation.

**Methods:**

Three focus groups were conducted among Black and Hispanic PLWH who use methamphetamine. Of the 13 participants, 5 had previously participated in a smartphone-based observational study of ART adherence and substance use. Discussants provided feedback on smartphone-based research, including receiving probes for HIV medication adherence, mood, and substance use as well as feedback on passive location-tracking for personalized messages. Transcribed audio-recordings were thematically coded and analyzed using the qualitative software MAXQDA.

**Results:**

Participants expressed confidentiality concerns related to potential unintentional disclosure of their HIV status and methamphetamine use and to possible legal consequences. They additionally expressed concerns around the invasiveness of daily assessments and the potential of methamphetamine use questions to trigger cravings. To mitigate these concerns, they suggested maintaining participant privacy by indirectly asking sensitive questions, focusing on positive behaviors (e.g., number of days sober), allowing user-initiated reporting of location to tailor messages, and ensuring adequate data protections. In addition to financial compensation, participants cited altruism (specifically, continuing a tradition of volunteerism in HIV research) as a motivator for potentially engaging in such research.

**Conclusions:**

Minority PLWH have concerns regarding the use of smartphones for ART adherence and methamphetamine sobriety intervention research. However, minority PLWH are likely to participate if studies include appropriate protections against risks to confidentiality and experimental harm and are designed to offer future benefit to themselves and other PLWH.

## Introduction

Consistent adherence to antiretroviral therapy (ART) is important, both for the well-being of people living with HIV (PLWH) and for public health efforts to curb the HIV epidemic [1]. Methamphetamine (MA) use, however, is common among men who have sex with men (MSM) living with HIV in Southern California and poses a significant barrier to ART adherence [2–4]. Indeed, population surveys suggest high levels of concurrence between HIV infection and MA use, with estimates of HIV infection ranging between 23 and 86% among MA-using MSM [5]. Furthermore, MA use has been associated with poorer ART adherence in cross-sectional and longitudinal research, with both decreased odds of ART adherence among PLWH who use MA compared to those who do not as well as decreased odds of adherence on a day that an individual uses MA compared to one without MA use [4, 6].

While MA and other substance use straddle racial and ethnic lines, the burden due to substance use is such that Black and Hispanic PLWH are, as a community, subjected to greater negative health and social consequences that overlay existing poorer outcomes in the HIV treatment cascade [7, 8]. For instance, although Black and Hispanic PLWH are no more likely to use MA than White PLWH, studies document worse ART adherence among Black and Hispanic PLWH in the context of MA and other substance use [9–12]. Such disparities in ART adherence and HIV-related outcomes are often described as developing from the syndemic, or the clustering of co-occurring epidemics, of HIV and substance use that results in mutually amplifying health problems, stoked by social and economic inequities [13].

The deleterious effect of active substance use on ART adherence can be attenuated by the provision of enhanced clinical services, for instance, through drug use disorder treatment and mental health counseling [14]. However, a number of factors, including sporadic visits, limited contact-time with patients, and poor patient insight into triggers of drug use can contribute to a framework that is inadequate for providing timely intervention to patients [15]. Furthermore, minority PLWH may experience additional structural and social barriers to seeking and receiving care, such as the unavailability of culturally responsive services and concerns about stigma [8]. Given the obstacles of providing effective clinical care, as well as the human and fiscal costs associated with active drug use in the context of HIV infection, novel and alternative methods for providing timely intervention are needed to help begin to ease the effect that MA use can have on ART adherence among PLWH.

Recent research on cellphone ownership indicates that the vast majority (77%) of US adults now own smartphones [16]. With the growing prevalence of smartphones and the rise of customizable applications for health promotion, smartphone-based interventions are increasingly able to dynamically assess risk for substance use and medication nonadherence and deliver flexible interventions [15]. Furthermore, given equivalent rates of smartphone ownership among ethnic minorities in the US (estimated between 75 and 77% among Blacks and Hispanics), new interventions may further equity in access to resources and clinical care [16, 17]. Specifically looking at PLWH and substance-using populations, preliminary evidence optimistically suggests that mHealth tools can be efficacious in supporting ART adherence and harm reduction in the context of substance use [17–20].

While mHealth approaches address many of the limitations associated with traditional quantitative methods and intervention, smartphones have extraordinary latitude for data collection, such that their use raises ethical challenges for researchers. In particular, concerns around beneficence, autonomy, informed consent, privacy, and data management arise because of the collection of vast amounts of highly personal data, the need to maintain privacy, and the heightened risk of disclosure of illegal behaviors [21–24]. Furthermore, studies examining the concerns of PLWH regarding their participation in mHealth research show that ethical concerns are not only harbored by researchers but also by their intended beneficiaries. Indeed, participants similarly cite concerns with privacy, risk of stigma, control over their own information, and trade-offs made during participation [25–28]. Despite this, determining the relative risk–benefit ratio of studies using novel technology has typically relied on the expertise of investigators and their institutional review boards, resulting in limited input from research participants [25, 29]. Consequently, this study was designed to engage Black and Hispanic PLWH with recent MA use in focus group discussions about the risks and benefits of research on the use of smartphones to promote ART adherence in the context of substance use.

## Methods

### Participants

Participants were recruited from the HIV Neurobehavioral Research Center (HNRC) and Family Health Centers of San Diego (FHCSD). Inclusion criteria for participation were (i) cisgender male reporting sex with men, (ii) age 18 years or older, (iii) Black race or Hispanic ethnicity, (iv) English-speaking, (v) having a diagnosis of HIV infection and a current prescription of ART, (vi) at least 1 day of self-reported ART nonadherence within the last 30 days, and (vii) self-report of MA use within the last 30 days. In order to generate robust discussion around research using a hypothetical tool to reduce MA use and increase the ecological relevance of feedback provided, potentially eligible participants were individuals with one or more self-reported previous attempts to quit MA use at some time in the past (e.g., by tapering MA use with the goal of cessation, participating in a substance use disorder program, or quitting “cold turkey”). Exclusionary criteria were visible signs of inebriation at the time of the study visit or inability to provide informed consent (e.g., due to active psychosis).

Thirteen individuals participated in three focus groups. One focus group was composed of non-Hispanic Black participants (*n* = 5), one of participants of Hispanic ethnicity (*n* = 3), and the third of Black and Hispanic individuals with previous participation in a pilot smartphone-based observational study of ART adherence and substance use (*n* = 5). Individuals with previous participation in the observational study (later referred to as the “expert group”) had experience responding to daily probes of their ART adherence, mood, and MA use, and they also had experience with location-tracking using global positioning system (GPS) devices—all features raised in the discussion of the hypothetical app to support ART adherence.

### Procedure

The study protocol was approved by institutional review board of the University of California, San Diego, and all participants provided informed consent after the nature of the study and possible consequences of participation were explained. Prior to the focus group discussions, participants completed a brief socio-demographic background questionnaire and standardized measures of ART adherence and substance use. Light meals and refreshments were provided and participants were compensated $40 for their time.

### Measures

#### Socio-demographic background questionnaire

In order to characterize the study sample, participants completed a brief background questionnaire assessing race and ethnicity, age, income, housing stability, and estimated duration living with HIV.

#### Antiretroviral therapy adherence

Adherence to ART was assessed using the 3-item Center for Adherence Support Evaluation (CASE) Index [30].

#### Severity of MA use

Severity of MA use was assessed using a modified version of the Drug Abuse Screening Test (DAST-10). The DAST-10 is a validated brief self-report instrument that yields a quantitative index of the degree of consequences related to substance abuse [31]. For this study, the DAST-10 was modified to ask specifically about problems related to MA use.

#### Readiness to change

Although all participants had prior attempts to quit or reduce their MA use, variability in their preparedness to reduce MA use was assessed using an adapted Readiness to Change Questionnaire [32]. The Readiness to Change Questionnaire assesses stages of behavior change according to Prochaska and DiClemente’s Transtheoretical model [33].

### Focus groups

Focus groups were held in a conference room located at the HNRC and lasted between 60 and 90 min. Participants were first oriented to the use of mobile phones for health behavior change in a brief discussion of apps that they were familiar with; following this, the notion of using an app in research to support ART adherence was introduced. A sequential approach was taken to verbally introduce and describe a hypothetical research smartphone app with multiple components that could assess ART adherence (and other relevant contextual factors) and also provide support for adherence in the context of MA use. Specifically, participants were successively asked about their opinions of participating in research using this hypothetical app to gather information about their (i) ART adherence, (ii) mood, (iii) MA use, and (iv) GPS location. Following discussion around assessment within each successive component of the app, participants were also asked to provide feedback on how the app might better support adherence in the context of substance use. Examples of questions that participants were posed included, “Please share your thoughts about providing information about your MA use using the smartphone app” and “How else would you want these questions to be asked?”

All focus groups were audio-recorded and transcribed verbatim without identifiable information. The discussion guides used in the focus groups were constructed with input from a community advisory board (CAB) in order to ensure respect for participants and relevance of questions. The CAB was composed of seven service providers and allies of the lesbian, gay, bisexual, transgender, queer (LGBTQ); Black; and Hispanic communities in San Diego; each member had varying levels of expertise in HIV, community advocacy, and substance use treatment or prevention. CAB members met with study personnel prior to the start of data collection to provide feedback on draft versions of the guide.

### Coding strategy

Subsequent to transcription, a thematic content analysis approach was carried out to identify emerging themes [34, 35]. Transcripts were independently coded by two investigators using the qualitative data analysis software MAXQDA [36]. A coding dictionary consisting of mutually exclusive code definitions and memos was constructed. Initial inter-rater reliability was low, resulting in further iterations of code refinement and assignment. Disagreement between coders in code assignment resulted in multiple rounds of review and coding and was resolved through the establishment of consensus. The final Cohen’s kappa coefficient, which considers the likelihood of the agreement between users occurring by chance, was high (> 0.9), indicating good inter-rater reliability [37].

## Results

### Participant characteristics

Table 1 summarizes participant demographic, ART adherence, and substance use characteristics. Collapsed across the three focus groups, five participants identified racially as Black, five as White, and the remainder as mixed race; the majority of participants were of Hispanic ethnicity (*n* = 7). The median age was 46 years and most participants reported being unemployed (*n* = 11) and with low incomes (< $10,000; *n* = 7). A substantial minority of individuals (*n* = 5) reported being unstably housed (living either outdoors, in a shelter, or at a treatment facility). With respect to HIV characteristics, participants reported living a median of 14.3 years with an HIV diagnosis and a median of 10 years on ART. The majority of participants (*n* = 9) reported poor ART adherence over the past month (CASE ≤ 10). With regards to substance use, about half of participants (*n* = 6) had scores on the DAST (DAST ≥ 6) reflecting a possible MA use disorder and the majority (*n* = 8) endorsed attitudes towards their current MA use consistent with precontemplation and contemplation of cessation.

**Table 1:** Socio-demographic and substance use characteristics of study participants

Characteristic	Total sample (*n* = 13)
**Demographics**	
Age, median [IQR]	46 [38, 52.5]
Race and ethnicity, *n* (%)	
Black	5 (39)
White	5 (39)
Mixed	3 (23)
Hispanic	7 (54)
Employed, *n* (%)	2 (15)
Income < $10,000, *n* (%)	7 (54)
Unstably housed, *n* (%)	5 (42)
**HIV-related variables**	
Years living with HIV infection, median [IQR]	14.3 [9.3, 18.5]
Years on ART, median [IQR]	10 [5, 19]
Poor ART adherence (CASE ≤ 10), *n* (%)	9 (69)
**Substance use**	
Stage of readiness to change (MA use cessation), *n* (%)	
Precontemplation	1 (7)
Contemplation	7 (54)
Action	5 (39)
Possible MA use disorder (DAST≥ 6), *n* (%)	6 (46)

*IQR* interquartile range, *ART* antiretroviral therapy, *MA* methamphetamine, *CASE* Center for Adherence Support Evaluation, *DAST* Drug Abuse Screening Test

### Themes related to concerns with research participation and risk mitigation

Table 2 provides summaries of opinions and representative quotations that emerged from discussions of components of the hypothetical app as well as suggestions brought forward by discussants to mitigate perceived risks.

**Table 2:** Themes and exemplar quotes related to concerns and barriers of using of the hypothetical app as well as suggestions to mitigate concerns and encourage participation

App feature	Concerns and barriers	Suggestions to mitigate concerns
1. Adherence messages	• **Unintentional disclosure of sensitive health information** “Just don’t say HIV. A lot of people aren’t comfortable with that… A lot of people, and I can only just speak for myself, but a lot of my associates don’t know. I’m pretty sure I would lose friends if they knew.” • **Potential low impact of adherence messages due to influence of peripheral factors** “I don’t know if—excuse me. I don’t know if an app would even help me because you send me a text sayin’, ‘Take your meds,’ if I’m doin’ meth and haven’t taken it, it’s like, ‘Oh, well.’ I still won’t take it because you have to take your meds with the food.”	• **Customize messages to individuals’ levels of comfort with disclosure** “… personalize it to different people. Some people are more private about their HIV status or AIDS status than others are.” • **“Code” reminders so that they are not directly about adherence** “…you wanna keep it confidential, they won’t know that it’s about or meds or anything. You see what I’m sayin”? “Something like, ‘You have a doctor’s appointment today.’ Then you confirm.” • **Provide messages related to factors that impact adherence and motivate other health behaviors** “Staying healthy and having that kind of self-esteem and things that go deeper than medication. That sort of thing… personal habits.”
2. Mood messages	• **Discomfort with divulging negative emotions to others** “I don’t know… Some reason, I just don’t want anybody to know that I’m goin’ through depression.” • **Discomfort with self-awareness of negative emotions** “Answering all those questions made me more aware of my feelings, and I didn’t necessarily like it…”	• **Simplify mood questions** “Mood wise, I would just keep it light, simple. Like… sunny or somethin’.” • **Provide content to elevate mood** “Probably something nauseating cute…like a little piglet wiggling its butt or something like that, just to cheer you up on the way.” “Create like an app where you can motivate at the same time… ‘you’re special, you’re a winner.’”
3. MA messages	• **Potential legal repercussions from disclosure of illegal activity** “Yeah. I’m gonna have to know what were you guys gonna do with that information because I’m home this weekend. If I got a question like that, and I was using at that time, there’s no way I’d wanna answer you that I’m using… Yeah. I just verified it, so come get me.” • **Triggering of cravings and meth use** “I’m more inclined to think that askin’ that question may cause some people who might be tryin’ to stop to relapse.” • **Impingement on personal autonomy** “It’s something personal, but still, it’s your decision to stop or not. We know that it’s not good to use that drug. We already know. We might have many motives to use it.”	• **Explicitly state nondisclosure to law enforcement during consent procedures** “To satisfy his paranoia, that you guys say in the contract or whatever that you’re not gonna call the police because you’re using meth, or any drugs.” • **Code questions to indirectly refer to MA use** “Have two faces. A good or a bad. Then just have those faces determine whether—meaning, did you use, or did you not use? … What color are you today? Then just pick—if you use that day, you just pick a certain color. It doesn’t have anything to do with anything connected to the law enforcement or whatever.” • **Direct attention to positive behaviors, e.g., duration of abstinence** “…days clean. Have a check mark where you can mark how many days you been clean. Thirteen, fourteen. Have you missed—if you’re not clean, just X or somethin’… use a more positive term.” • **Advocate harm reduction** “It would be a good tool, at least to avoid those skipping medications that I have when I use drugs, if I decide to keep on using drugs… it reminds you right away that you have to take care of yourself, even you didn’t take care of yourself because you were using the drug, right?”
4. Location assessment	• **Potential for legal repercussions** “We won’t have to be going to jail because of what we were honest about, or connected to, in the research study… Well, as a result, boom, you’re charged possession and whatever… People worry about these things.” • **Invasiveness of continuous monitoring** “It’s like having a camera in every corner in every alley.”	• **Allow self-reporting of movements and tailor messages accordingly** “Maybe you could change it, word it as such, ‘Steppin’ out? Don’t forget to pack fun pack or whatever.’ That way, if they’re leavin’ and goin’ somewhere, I need to make sure I take this with me.”
5. Overall study features	• **Potential for inappropriate timing of assessments** “When I’m using, I don’t answer my phone, period. The ringer’s down. I don’t wanna hear it ring. I don’t wanna hear it buzz… that brings my high down, then I have to get high again, just to get where I was before.” • **Participant burden with repeated assessment** “…when you pushin’, then I be like, ‘Who you?’ … When it’s comin’ to me like that, I’m like, ‘Forget you.’ That put me, more or less, in a bad mood, and I won’t even bother to answer the question.” • **Benefit the study and contributions to knowledge** “It’s a no-brainer. Drugs get in the way. They do. That’s period.” • **Inequitable access to smartphones and data plans** “I don’t think the app—not everybody pays their cell phone bill on time, so they get cut off or, especially if they’re reliant on an Obama phone, they’re limited to two gigabytes of data, which gets used up quickly with a movie. Then they can’t go online to do that.” • **Maintaining privacy on phones** “I’ve got a Smartphone and an Obama phone, and my friend who’s down and out, to get him back on his feet, I loaned him my Obama phone, and he sold it for food… The phones are traded commodities out there… Yeah…In fact, it passed through five hands before I found out who had it.”	• **Allow “snoozing” of questions** “What if they had a question that you could check the box, where you could answer later…?” • **Allow retrospective reporting of use** “Catering the survey for your needs would be something like the day after, or it’s three days later, and then they do a retrospective survey.” • **Increase autonomy in deciding levels of participation** “What if you could enable something like that or disable it, so you can use it sometimes, but if you’re like, ‘Okay, I find this too much,’ so you can turn on or off?” • **Increase variability and diversity of content** “You want the app to appeal to people and not turn them away. You gonna wanna have a lotta customization in it… it’s designed this way so they would look forward to goin’ into the app, maybe participating in things that they like. You might have several different things they could participate in on there… It’s like, ‘Let me see what they have to say today?’ I’m gonna look at it whether I take it [ART] or not.” • **Provide study phones with data plans** “… you can — not to bring up the other doctors, but Dr. [HNRC researcher’s name], you can do his study for nine months, eight months, and you get an iPhone… They tell you to use it for, the study…That might be helpful to someone.” • **Bolster app security** “I like the idea of an app with a sign-in because phones get lost all the time. Usually, they’re stolen by your friends who know your access code to get in the phone.” “…you know the information is encrypted or double encrypted and all that kind of stuff… give one—the security, the sense of security to answer a personal question.” • **Clear consent language and periodic reconsenting** “Ask permission. Do we have permission to check on your sobriety, yes or no? Maybe pose the question again. Could we ask you in 30 days? How about 90 days? Somethin’ like that. Permissions for everything that you might wanna do on that app, but not too many because then it becomes intrusive.”

#### ART adherence messages and assessments

Overall, participants held favorable opinions about receiving ART reminders through an app*—*“That’ll prompt somebody to take it at night if they forgot in the morning.” However, a primary concern with receiving probes and reminders for ART adherence was of the potential disclosure of HIV status to other people. Apprehensions of unwanted disclosure were high as participants reported concomitantly maintaining privacy about their HIV status and often sharing their personal phones or using them around “nosey” individuals (Table 2). To ameliorate the risk of unwanted disclosure, participants suggested that adherence messages exclude terms such as “HIV” or “medication”, depending on individuals’ levels of comfort with disclosure of a health condition. Furthermore, they suggested that messages be “coded” so that only the intended recipient would understand the meaning of the ART adherence questions and reminders (Table 2). Participants additionally expressed concern that receiving messages solely related to taking ART might have a low impact on improving adherence in the context of peripheral barriers influencing adherence (e.g., the need to take with food). Thus, they suggested that messages also emphasize overall health and wellness (Table 2).

#### Mood assessment and messaging

Participants raised concerns regarding responding to questions inquiring about their mood, as they described difficulty with divulging their negative emotions to other people and unease with revealing the presence of depressed mood. Furthermore, some individuals in the “expert” group reported developing greater self-awareness of their affect through daily reflection in the previous study and subsequent discomfort with knowledge of their negative mood states (Table 2). Given this, participants suggested that distress resulting from disclosure of difficult emotions and from introspection could be ameliorated by presenting simplified and brief mood assessments, e.g., by presenting a choice of emojis (in a manner similar to a visual-analog scale). Furthermore, in addition to merely assessing mood, participants suggested that the app provide content to elevate mood (e.g., inspirational quotes, affirmations) (Table 2).

#### MA assessment and messaging

Regarding the assessment of MA use, participants’ primary concerns were of the potential for legal consequences if details of their MA use were to fall into the hands of law enforcement officers. They additionally expressed concern that family members and friends, currently in the dark about their substance use, might become aware of their MA use. Participants were also worried that asking about MA use would potentially trigger cravings, stultifying efforts to maintain sobriety among those in recovery (Table 2). Given these concerns, participants suggested that the assessment of substance use be indirect and coded (for instance, through the use of colors—one indicating recent substance use and another indicating abstinence—or through the use of emojis). Additionally, they suggested that substance use questions direct attention towards positive behaviors (e.g., by assessing the number of days clean). Furthermore, participants suggested that the app de-emphasize cessation from substance use and promote harm reduction by providing reminders for adherence regardless of recent MA use (Table 2). Lastly, in order to assuage the concerns of future research participants, discussants reiterated the importance of stating clearly, in consent procedures and documents, that information about substance use would be protected from law enforcement*.*

#### Location assessment

Passive assessment of location was generally perceived unfavorably, even by participants with experience wearing GPS devices. Concerns with continuously providing location were that it was overly invasive, often described as “Big Brother”, and carried the risk for arrest and legal consequences if information about location and MA use were conjointly obtained by law officials (Table 2). Thus, alternatives to continuous assessment with lower perceived risk were discussed. Participants reported willingness to periodically respond to probes for location or to initiate location reporting, through a process like “checking-in” or providing a “snapshot in time” of their location, and subsequently receiving tailored adherence messages (e.g., reminders to carry extra medication on their person when leaving home) (Table 2).

#### Overall study features

In addition to concerns raised of specific components of a potential app, participants discussed issues related to the overall study design and with the maintenance of data integrity. With regard to study methods, participants reported concern that daily assessment could be intrusive and annoying, particularly if reminders or questions were sent at inconvenient times or repeatedly sent during periods of intentional non-responding (Table 2). Given these concerns, they suggested features to allow postponing responding by “snoozing” the app and that the app be adaptive by reducing the frequency of messaging during periods of voluntary non-response. They also suggested that the app allow for forms of retrospective responding—features that participants analogized to “blogging”—to capture events during “blackout” periods. Thus, participants advocated for greater autonomy in the times and ways that potential users would be able to interact with the app and provide information in order to alleviate the burden of responding to repeated probes for information. To reduce boredom and fatigue and to promote greater personal usefulness, participants also suggested personalizing the app to the individual and having significant variability in content. Specific customizations participants suggested were the ability to receive reminders for other medications, weblinks to health resources (e.g., to TheBody.com), and the ability to connect with other PLWH through the app and find social support (Table 2).

Focus group discussants also raised broader questions regarding the merit of the proposed research and of its fairness. In particular, participants pondered the benefit to society of intensive longitudinal research on ART adherence in the context of the MA use and the degree to which it would contribute to knowledge, beyond what is already known from prior studies. They also highlighted concerns around the potential for inequitable access to opportunities for research participation, as not all individuals have smartphones or sufficient data plans (Table 2). Thus, to improve the opportunities for all eligible individuals to engage in mHealth research, participants suggested that research studies provide phones and data plans for those who may need them (Table 2).

Finally, with regards to security of the app and maintenance of privacy, participants’ primary concerns were of access to responses to sensitive questions by other individuals in physical possession of their phones, as they indicated significant sharing of personal phones. To ameliorate this risk, participants suggested that the entire app be password-protected and that measures to allow secondary authentication are considered (such as verification of passwords via text-message or email). Additionally, to prevent external hackers from remotely gaining access to user information, participants suggested layers of encryption of data and visible signs of establishment of a secure connection (such as the image of a lock when an internet connection is secure) (Table 2).

### Themes related to motivators and perceived benefits of engaging in research

Despite endorsing multiple concerns, most participants (*n* = 11) indicated willingness to participate in a study utilizing the hypothetical app. Financial compensation was reported as a reason to participate; however, altruism was most-frequently cited as a motivator for willingness to participate. In particular, participants expressed the desire to contribute to a tradition of PLWH volunteering in HIV/AIDS research and a desire to help younger PLWH—“People have done this research for me before to help me get on the medications that I’m on now, and if they hadn’t of done it, I wouldn’t be where I am today, and so I come in there with that in the back of my mind, that I need to come in here and be totally honest with you, so that we can help the younger people.”

Of interest, participants indicated greater willingness to contend with the discomfort of answering invasive questions in the context of research rather than in supporting their own adherence as they weighed potential benefit to research and society above their own individual well-being—“If you’re part of a research study, let’s say, where, in fact, whether you’re on meth or when you last smoked it, that kind of thing… then you know that it’s feedback that you’re providing that’s gonna go to something. If you’re just wanting to ensure higher level of adherence in general, I don’t think that would be a good question to answer.”

Despite overall consensus of valuing societal over individual benefit, some participants reported anticipating developing personal insight and gaining coping skills—“... it hurts me to think, oh, god, when I answer those questions. How many times a day do you use? Every day, or always, [laughter] things like that, but it’s nice because it gives me that freedom of maybe I could find a solution or something that works better. I feel motivated that I might get something…. I feel like I might be able to get something positive to keep going in the right direction.”

## Discussion

MA use is a significant barrier to ART adherence and it contributes to disproportionate health burdens among minority PLWH [2–4]. Smartphone-based apps are a promising mode for delivering ART adherence and relapse prevention interventions; however, the acceptability of app-based interventions that collect and monitor highly sensitive information has not been sufficiently assessed among vulnerable individuals characterized by multiple stigmatized identities. Thus, the primary goal of this study was to explore the viewpoints of minority PLWH who use MA on the using smartphone-based interventions in research to facilitate ART adherence.

Overall, participants indicated willingness to engage in similar mHealth research to support ART adherence in the context of substance use, citing financial compensation and a desire to contribute to a tradition of volunteerism in HIV research. Altruism has previously been identified as a motivator of participation in other forms of HIV research (particularly in HIV cure studies) and the continued thread of altruism as a motive force for participation in this context suggests that PLWH may value contributing to society across a range of research areas [38–41].

Concomitant with their willingness to participate were concerns of the potential negative consequences resulting from research participation. Consistent with previous studies, participants in our focus groups described user-concerns about the privacy and security of sensitive information [25–27, 42, 43]. Given these concerns, participants suggested ways to ameliorate the potential harms and limitations they perceived. Specifically, they suggested phrasing questions for privacy and for easier self-reporting, tailoring the app to allow user-initiated reporting of location, and enhancing data protection methods. They also raised concerns of experimental harm that have been less frequently voiced in participatory research on mHealth technologies, including concerns about the potential for intrusiveness and burden due to repeated daily assessments, the potential for MA questions to trigger cravings, and the potential for questions about mood to induce negative affect. To limit negative repercussions of participation, discussants suggested coding and simplifying questions (for instance, using colors or emoticons) and also assessing positive behaviors (like number of days without MA use) as alternatives to frank inquiries about sensitive topics. Thus, in addition to attending to privacy concerns, developers of mobile ART adherence interventions are also tasked with considering ways of mitigating the potential psychological harms and discomforts that may result from frequent collection of highly personal self-reports of behavior or emotions.

Participants also acknowledged that, despite widespread phone ownership, studies involving the use of smartphones may inadvertently perpetuate existing inequities, as smartphone ownership is not universal nor are all individuals with phones able to consistently afford data plans. Thus, participants made considerations of fairness in the distribution of research opportunities and in the later diffusion of benefit from mHealth interventions. This concern is particularly relevant among PLWH who also use MA, as they experience lower socioeconomic status and higher rates of homelessness than PLWH who do not use MA [44, 45]. Indeed, in this sample, approximately half of study participants reported being of very low income or currently encountering housing instability. Given the potential for exclusion, providing study phones (that participants are able to keep them at the end of the study) and connecting participants to federally sponsored programs that provide cell phone services may help to limit the degree to which mHealth approaches overlay upon existing disparities [46, 47].

Lastly, across all components of the hypothetical app, participants reiterated the importance of personalizing their experiences. However, the high degree of customization and responsiveness to user input proposed by participants suggests a dynamic and personalized intervention that would be a challenge to systematically evaluate using traditional methodologies. Indeed, randomized clinical trials (RCTs), the gold standard for establishing efficacy of interventions, typically evaluate the efficacy of static treatment packages in order to maximize internal validity. But, to effectively and adaptively provide support, more novel research designs are required to evaluate app-based research. Consequently, factorial trial designs, such as the multiphase optimization strategy (MOST), the sequential multiple assignment randomized trial (SMART), and the micro-randomized trial, have been proposed as alternatives to traditional RCTs in mHealth research. These contemporary methods for designing and testing adaptive interventions are now used more frequently to evaluate interventional apps by identifying features for inclusion/exclusion, empirically determining tailoring variables and decision rules governing response in adaptive intervention, and incorporating randomized experimentation to facilitate valid inferences [48–51]. Taken together, recent methodological advances in trial design may facilitate the development of mHealth interventions that are better optimized to provide support to idiosyncratic participants who are couched within changing contexts.

In summary, limited previous work has included research participants in formative study phases; thus, a strength of this study was to engage representative individuals in discussions around the potential risks of participation in mHealth research and to obtain user-suggested feedback on ways of ameliorating those risks prior to implementing mHealth interventions. Findings from this study suggest that if participants perceive appropriate protections against risks to privacy and confidentiality, limited harm resulting from research participation, and future benefit to themselves and other PLWH, they are willing to be volunteers in mHealth research. They also reiterate the complexity of processes that contribute to judgments of risks and benefits in research studies; thus, incorporating participant perspectives may help to inform researchers’ and IRBs’ understandings of the relative risk–benefit ratio in studies that use novel technology. Additionally, integrating participant-informed measures against risks to privacy, confidentiality, and experimental harm may increase participant involvement and adherence throughout the course of research studies. Furthermore, as many of this study’s findings are applicable to mHealth research among PLWH, regardless of their race/ethnicity, this study may inform the development of ART adherence research among PLWH who use MA more broadly.

### Limitations

There are a number of limitations to this study. First, although attempts to engage a larger sample of participants were made, the sample sizes within the three focus groups were relatively small due to a high no-show rate. Additionally, the relatively stringent eligibility criteria (in particular, MA use within the last 30 days of screening) resulted in a smaller pool of eligible individuals for recruitment among a much larger population of MA-using MSM living with HIV. Future studies should attempt to recruit a larger sample of individuals, with greater variability in their MA use patterns, using multiple recruitment strategies in order to improve generalizability and promote sampling of a wider range of perspectives. Additionally, as a consequence of recruitment of only individuals with previous attempts to quit MA use, our study findings may not be as relevant for informing ART interventions among individuals who have never considered reducing their MA use. Furthermore, discussions in these focus groups revolved around the features of a hypothesized app. Thus, the feedback we received may only approximate the rich participant feedback that might be obtained in usability tests. Therefore, future research in mHealth intervention development should iteratively solicit feedback at multiple interval stages of app development to improve acceptability and user experience.

## Conclusions

Our findings offer contributions to the literature on designing mHealth interventions in the context of HIV and substance use by exploring the perspectives of minority PLWH who use MA. Consistent with principles of participatory-based design, we solicited input relevant to identifying and remediating risks that may be associated with participation in mHealth interventions among substance-using PLWH [52]. Future research on app-based interventions will likely benefit from greater inclusion of diverse research participants as stakeholders who are integrally involved in the study design.

## Data Availability

The datasets generated and/or analyzed during the current study are not publicly available due to privacy and confidentiality reasons; however, deidentified transcripts are available from the corresponding authors upon reasonable request.

## Abbreviations

ART: Antiretroviral therapy
HIV: Human immunodeficiency virus
AIDS: Acquire immunodeficiency syndrome
PLWH: People living with HIV
MA: Methamphetamine
MSM: Men who have sex with men
mHealth: Mobile health
HNRC: HIV Neurobehavioral Research Center
FHCSD: Family Health Centers of San Diego
GPS: Global positioning system
CASE: Center for Adherence Support Evaluation
DAST: Drug Abuse Screening Test
CAB: Community advisory board
LGBTQ: Lesbian, gay, bisexual, transgender, queer
IQR: Interquartile range
RCT: Randomized controlled trial
MOST: Multiphase optimization strategy
SMART: Sequential multiple assignment randomized trial
